# Psychiatrists and neuroscientists of Indian origin in Canada: Glimpses

**DOI:** 10.4103/0019-5545.69213

**Published:** 2010-01

**Authors:** Amresh Shrivastava, D. Natarajan

**Affiliations:** Department of Psychiatry, The University of Western Ontario and Associate Scientists, Lawson Health Research Institute, London, Physician-team Leader, Early Psychosis Program, Regional Mental Health Care, St. Thomas, Ontario, Canada,; 1 Department of Psychiatry, 2110 Hamilton Street, Third Floor Regina, SK, S4P 2E3, Saskatchewan, Canada

**Keywords:** Indian, Psychiatrists, Canada

## Abstract

Psychiatrists of Indian origin are popular in Canada, being firmly rooted in the Canadian mental health system, and they have been making considerable contributions internationally. The Indian Psychiatric Society has long been collaborating with and inviting contributions from overseas Indian psychiatrists, particularly those in academics, and this collaboration has fructified well. There are several different challenges these psychiatrists have had to face in their own specialty work, with having to adjust to a new culture, new ways of living, and new ways of work. Our colleagues of Indian origin have demonstrated excellence in almost all fields of mental health and neurosciences. There are many popular teachers, outstanding researchers, and psychiatrists in community practice and community development. The Early Psychosis Program, Mood and Anxiety Program, Perinatal Psychiatry, Women’s Mental Health, and Postpartum Mental Health are some of their key areas of research. Our basic scientists are involved in experimental design, neurochemistry, imaging, and genetics, where they have made their mark with acclaim. This article highlights some of the achievements of a few members and is by no means completely representative of the entire work that psychiatrists of Indian origin are doing in Canada, providing readers with a glimpse of our labors away from home.

## INTRODUCTION

Psychiatrists of Indian origin are popular in Canada. Concerns of international mental health and the contribution of our psychiatrists abroad has been acknowledged since long.[1] International mental health is progressing and physicians from all countries are making efforts for collaborations in service, education, as well as research,[2] and Indian psychiatrists are firmly rooted in Canadian mental health care. They have become a part of the global power, making significant contributions toward international mental health. The knowledge and experience of Indian psychiatrists have given them a certain unique edge required to exceed expectations within the working norms of the field of psychiatry.

Their commitment and integration in academic and clinical work has been absolute. No one thinks of psychiatrists from Indian origin as aliens. As in very many other countries, psychiatrists of Indian origin have excelled in Canada. They have gone the extra mile and have been conscientious about their roles and responsibilities.

Although the term ‘International medical graduates’ and ‘Immigrant psychiatrist’ do exist, they have become somewhat irrelevant. Psychiatrists from India now share responsibilities in all fields of clinical care, service development, educational research, management, and administration, and the Indian Psychiatric Society has encouraged partnership with our fellow professionals abroad.[3–4] Indian scientists are contributing in basic sciences as well as in clinical medicine in Canada.[5]

Adjusting to a new country, to a new culture, new ways of living, and new ways of work are obvious, but daunting challenges. The health services, organization and system management is very different and unique. In Canada, it is neither akin to the British form (a system many from the Indian origin are exposed to) nor anywhere close to the Indian system. Many new skills have to be freshly learnt and some skills have to be literally unlearnt. Cultural adjustment for the professionals’ families and children has been another area of challenge, besides the severely cold and snowy winters.

Although the government, the licensing body, and the people of Canada had been welcoming, receptive, and generous, our doctors had to go a long way in order to prove themselves. They had to work on adapting skills desired for the local conditions. Like in any other country, an immigrant physician needed to have a license to practice. They had to undergo the local training and pass the examination of the Royal College of Psychiatrists of Canada and obtain the license from the provincial licensing body from the state in which they desired to practice medicine. Regrettably, the Indian qualification and residency programs, even those from some prestigious institutes and universities, were not sufficient qualification, although this very experience has often been the backbone of the Indians’ effective performance.

As per my information, the presence of psychiatrists of Indian origin goes as far back to between five and six decades. The land of Canada became a preferred destination for mental health professionals from several parts of the world. Some of them came after their undergraduate education and some with postgraduation. A majority of them traveled to Canada after working in the UK or Ireland, and obtained a membership of the Royal College of Psychiatry, UK. Some of them came to work here from east African countries and from the Caribbean islands. In this description I will briefly touch upon the achievements of some, as known to me [There are likely to be omissions and this is inadvertent, and regretted].

One of the first ones to arrive was the late Jambur Anant. I had the pleasure of interacting with him closely on several occasions in the United States, the place where he subsequently moved from Canada. Prof. Anant worked in field of psychopharmacology; he was an acclaimed scientist and a good administrator. His significant contribution in understanding the treatment with atypical antipsychotics has been noteworthy.

Vivek Kusmakar was another distinguished psychiatrist of Indian origin. He came to work in Canada and subsequently moved to the United States. He too worked on the safety and tolerability of antipsychotics, problems of adolescence, depression, and psychoneuroendocrinology. Vivek and Jambur both visited India several times and participated in academic meetings. Their interest, intellectual insight, and vision for psychiatry had made them very good contributors to mental health. Both of them were our guests during the World Psychiatric Association section meetings at the ‘International Convention of Biological Psychiatry’ organized by the Indian Psychiatric Society section of Biological Psychiatry in Mumbai, in 1996.

One of their widely recognized acievements is the establishment of the section of ‘Indo-Canadian Psychiatry’ in The Canadian Psychiatric Association (CPA), and independent of this, Indian psychiatrists in Canada also have their own professional body, ‘The Indo Canadian Psychiatric Association’. The latter was founded by the dedication and hard work of Aruna Thakur, Dhanapal Natarajan (the founding President), and Chinnapalli Manjunath. Savalai Manohar currently chairs it. Aruna Thakur also established the section of Cultural Psychiatry when she was the President of CPA. Soma Ganesan leads the Cultural Psychiatry division in Vancouver.

Interaction with professional bodies and licensing authorities in various provinces is the starting point of a professional career that begins all over again. It is heartening to note some of the Indian psychiatrists achieving the highest prestigious posts in the field. Raidu Koka, a distinguished teacher and administrator, worked with a number of professional organizations and served on several committees of the College of Physicians and Surgeons of Ontario (CPSO). He was the president of the CPSO in 2008 – 2009. I count this as a singular distinction of great honor, which one can achieve in this specialty. During his tenure he brought about important changes in the rules of the college, which proved useful to the physicians.

Noteworthy achievers in the administration of the Departments of Psychiatry include Prasada and Shubhash Jain. Prasada chaired the department of psychiatry at the University of Western Ontario (UWO), and Jain chaired the Department at the University of Newfoundland. Both of them have been very popular and successful leaders.

The next important area is the service to the community and to patients. A majority of our psychiatrists are involved exclusively in direct patient care: either in private practice or in community hospitals. They share the burden of providing services to a large number of patients. Some have, besides the routine practice of psychiatry, also been involved in community development programs, while working with non-profit organizations.

Psychiatrists in teaching institutions have alsoto made their mark in their multiple roles of teaching, education, research, clinical service, and program development. Some of them also have to undertake the job of administration and management, while doing all of these. It is difficult to clearly classify the roles and achievements of these physicians. I will try to highlight their predominant contributions:

Canada has invested hugely in research in basic sciences and public health, with a vision to cope with the demands that will rise over the next 20 years. Psychiatric services in this country are program-based. Important programs, which have been established and have become exemplary, are the programs for ‘early intervention and prevention of psychosis’ and ‘mood and anxiety disorders’. A number of Indian researchers are leaders and are being highly acclaimed for their work in the program.

Research in ‘schizophrenia and related disorders’ is one of the many such study-based programs. Some important leaders and contributors in this area have been: Ashok Malla, Kshitij Kapur, Rohan Ganguli, Rahul Manchanda, Raj Harricharan, Amrindar Singh, S Rai. Significant contribution has also been made in the field of basic sciences relevant to schizophrenia, for example, genetics, experimental design, animal studies, and neurochemistry. Some of the leaders in this field are Ravi Menon, Shiva Singh, RK Mishra, AK Tiwari, M. Mistry, RD Jindal, and Lalit Srivastava. These scientists have made outstanding contributions in experimental design and neuroimaging research in mental disorder. Menon who works in the field of imaging and neuroscience at the prestigious Robert Research Institute deserves special mention.

Kshitij Kapur’s study is considered exemplary in neuroimaging, particularly his researches in the dose-response relationship of antipsychotics and dopamine blockage, the role of dopamine in psychosis, and related neurobiological studies using positron emission tomography (PET), besides conventional imaging.[6]

In the field of clinical research, early psychosis and first episode schizophrenia have been priority areas for Indian psychiatrists in Canada. Our researchers are international leaders in establishing specialized programs, and the important findings emerging from these studies are worth mentioning. Conceptually, ‘there is more to early intervention that intervening early’ is a path-breaking concept of Ashok Malla.[7,8] Researchers here have worked on the significance of the duration of untreated psychosis, psychosocial interventions, the treatment of comorbid conditions, treating substance abuse for maximizing benefits of early intervention in early psychosis, early identification models, and effectiveness of long-acting antipsychotics. Other interesting work includes formulation and testing of the hypotheses for the outcome inat two years, and in the long run, indicating the cost-effectiveness of a specialized program by the end of five years.. These excellent research findings are the result of the Early Intervention Program.

Early intervention in psychosis is one of the priority areas of service and research for the government of Canada. Canada is among the first few countries to establish the program for early intervention and prevention of psychosis, which was more than 12 years ago. This program has delivered service to a large number of patients and has contributed significantly to the early intervention research database. Ashok Malla is a distinguished international leader in the field of early intervention; he has to his credit the establishment of two early intervention programs, one at UWO and another at McGill University, Montreal.

The Prevention and Early Intervention Program for Psychoses (PEPP) at the UWO, London, is a community-focused Mental Health Program, which provides prompt assessment and is a comprehensive, phase-specific, medical and psychosocial treatment for individuals experiencing their first episode of psychosis. Rahul Manchanda, a distinguished researcher in this field is the current director of this program in London.[9,10]

Rohan Ganguli is a man with a very good international reputation for conducting innovative research to understand and mitigate the factors that cause premature death and disablement. He was one of the first scientists to point out the relationship between new antipsychotic medications and excessive weight gain. He has conducted scientifically rigorous interventions to reduce this adverse effect of the medications. Ganguli has recently moved from Pittsburgh to join as the Vice President of Research, at the premier institute of the Center for Addiction and Mental Health (CAMH), at the University of Toronto. He is likely to build on his research program by investigating non-psychiatric health issues in individuals suffering from schizophrenia and other serious and persistent mental disorders. His research goals are to increase the effectiveness of risk-reducing behavioral interventions, to prevent the development of risk factors for cardiovascular disease, and to explore the role of inflammation in obesity associated with schizophrenia.[11–13]

Mood disorders are another preferred area of psychiatric research in Canada and a number of our clinical researchers are involved in this; Laxmi Yatham, Shaila Misiri, Virendar Sharma, and Sagar Parikh, to name a few. Yatham and Sharma work in the area of bipolar disorder. Significant research outcomes have been 5-HT2 receptor, brain serotonin electroconvulsive therapy, neurocognitive fiction in mania, psychopharmacology of bipolar disorder, side effect of antipsychotics, particularly weight gain, and the course, outcome, and guidelines for management are some of the important areas of interest.[14,15] Yatham and Sharma have started working more recently on the area of post partum depression and their observation that ‘Post partum depressions are severe in the first few weeks and most of these are of bipolar nature’ has been well received. Through a number of articles Yatham has highlighted the need for screening, diagnosis, training, and treatment of this condition. His second theme, ‘there is a diasthasis of bipolarity in treatment-resistant depression,’ is also a new concept for further investigation. He is also interested in the psychopharmacological management of bipolar disorder.[16,17] Parikh is another researcher who has made a mark in this field; his area of interest mainly being neurochemistry, genetic polymorphism, socio-demography, and management issues. All three researchers have been actively involved in developing the Canadian Network for Mood and Anxiety Treatment (CANMAT) guidelines, to manage patients with bipolar disorder, which is quite popular in Canada.[18]

In the field of mood disorder, Shaila Misri, who is one of the leading reproductive psychiatrists in North America, and who is internationally recognized as a pioneer in women’s mental health and reproductive issues has established a very important program on women’s mental health, particularly dealing with prenatal psychiatry. She is the founder and director of Reproductive Mental Health at both St. Paul’s Hospital and BC Women’s Hospital, as well as the Health Center in Vancouver. Canada[19–21]

Kiren Ribaro has been active in geriatric psychiatry, and Soma Ganesan in transcultural psychiatry. Our research faculties are also involved in the work of reviewing grants, collaborations, and research administration. All these researchers have a track record of grantmanship. They are successful candidates in obtaining grants from many prestigious funding organizations including the Canadian Health Research Institute.

Praful Chandrana is a national faculty for training in psychiatry. He has been a member of a number of education communities in the Royal College. He has been the director of postgraduate education in the UWO. A number of students look up to him for guidance and he is a ready resource for each and every student of psychiatry in Canada, in the field of education.

Many Indo-Canadian psychiatrists are leading experts on teaching of psychiatry to Canadian Medical students and Residents. Sagar Parikh is the Director of Continuing Mental Health Education at the University of Toronto. Raj Harricharan is the Director of Undergraduate Education and Virendar Dua is running the Continuing Medical Program at UWO. Jatindar Thakar is the Vice Dean of the Continued Professional Development Department of UWO. Similarly, a number of our colleagues occupy commendable positions and lead programs in the Department of Continuing Medical Education, Undergraduate and Postgraduate Education, as well as in Faculty Development. I have only mentioned a few.

Each one of those mentioned herein is a recipient of many distinguished awards in research and education. Many of them have been awarded the Teacher of the year award for their contribution to teaching. Some of them have been awarded the Fellow of the CPA awards for their contribution in a variety of areas of mental health, including teaching.
